# Delivery of Nanoparticle-Based Radiosensitizers for Radiotherapy Applications

**DOI:** 10.3390/ijms21010273

**Published:** 2019-12-31

**Authors:** Francis Boateng, Wilfred Ngwa

**Affiliations:** 1 TIDTAC LLC, Orlando, FL 32828, USA; 2 Department of Physics and Applied Physics, University of Massachusetts Lowell Lowell, MA 01854, USA; 3 Department of Radiation Oncology, Brigham and Women’s Hospital, Boston, MA 02115, USA; 4 Department of Radiation Oncology, Harvard Medical School, Boston, MA 02115, USA

**Keywords:** nanoparticles, radiosensitizers, radiotherapy, brachytherapy, smart radiotherapy biomaterials, eluting-spacers, in situ delivery via implants, cancers, reoccurrence

## Abstract

Nanoparticle-based radiosensitization of cancerous cells is evolving as a favorable modality for enhancing radiotherapeutic ratio, and as an effective tool for increasing the outcome of concomitant chemoradiotherapy. Nevertheless, delivery of sufficient concentrations of nanoparticles (NPs) or nanoparticle-based radiosensitizers (NBRs) to the targeted tumor without or with limited systemic side effects on healthy tissues/organs remains a challenge that many investigators continue to explore. With current systemic intravenous delivery of a drug, even targeted nanoparticles with great prospect of reaching targeted distant tumor sites, only a portion of the administered NPs/drug dosage can reach the tumor, despite the enhanced permeability and retention (EPR) effect. The rest of the targeted NPs/drug remain in systemic circulation, resulting in systemic toxicity, which can decrease the general health of patients. However, the dose from ionizing radiation is generally delivered across normal tissues to the tumor cells (especially external beam radiotherapy), which limits dose escalation, making radiotherapy (RT) somewhat unsafe for some diseased sites despite the emerging development in RT equipment and technologies. Since radiation cannot discriminate healthy tissue from diseased tissue, the radiation doses delivered across healthy tissues (even with nanoparticles delivered via systemic administration) are likely to increase injury to normal tissues by accelerating DNA damage, thereby creating free radicals that can result in secondary tumors. As a result, other delivery routes, such as inhalation of nanoparticles (for lung cancers), localized delivery via intratumoral injection, and implants loaded with nanoparticles for local radiosensitization, have been studied. Herein, we review the current NP delivery techniques; precise systemic delivery (injection/infusion and inhalation), and localized delivery (intratumoral injection and local implants) of NBRs/NPs. The current challenges, opportunities, and future prospects for delivery of nanoparticle-based radiosensitizers are also discussed.

## 1. Introduction

Nanotechnology has unlocked and broadened cancer diagnostics and the therapeutic window of radiotherapy, resulting in the prospect of nanoparticle-based radiotherapy with the aim of increasing radiotherapeutic efficacy [1,2,3]. Current radiotherapy (RT) modalities used in cancer treatment are employed in over 50% of cancer patients, and RT may be combined with other treatments such as surgery or chemotherapy [4,5,6,7]. Damage to tumor cells is relative to the energy (dose) delivered [3]. However, since RT consists of delivery of high intensity ionizing radiation to the tumor for the purpose of killing tumor cells, this can lead to damage of the surrounding healthy tissues [8,9,10]. Most RT procedures (especially, external beam RT) can damage or injure (break DNA of healthy cells), or cause cell death (kill healthy cells). The damaged or injured cells can recover (repair, and function as normal cells), die (cell death), or be unable to repair properly, resulting in DNA mutation and potential cancers. For instance, it has been reported that some patients undergoing RT treatment are likely to have radiation-related risks, such as cardiovascular complications, radiation pneumonitis, development of secondary cancers, and lymphedema [11,12]. If the RT dose delivered does not kill all the cancer cells, they will regrow in the future. Distant cancer cells or any cancerous cell that received a low dose (a nonlethal dose) of radiation can develop resistance to radiation, which subsequently require elevated doses; which, in the end, results in death of the surrounding normal tissue [8]. 

Despite advancements in RT modalities over the past decades due to the progress in engineering and computing, tumor radiation resistance and toxicity to healthy tissues, among other inherent flaws in RT, still make it necessary to consider the advantages and disadvantages of RT [4,8,13]. Many attempts to increase RT efficiency while decreasing the physiological disadvantages have been investigated, including radiosensitizing tumor cells via nanoparticles (NPs) [3,8], enhancing the radiation resistance of normal tissues, and reversing the radiation resistance of tumor cells [8,14]. The approach of using NBRs/NPs for radiosensitization of cancerous tumors to enhance the radiotherapeutic ratio is very important. However, challenges, such as the choice of NBRs/NPs, the route of delivery of the NPs, and lowering the negative side effects on normal tissues, need to be overcome [2,4,15]. 

**Figure 1:**
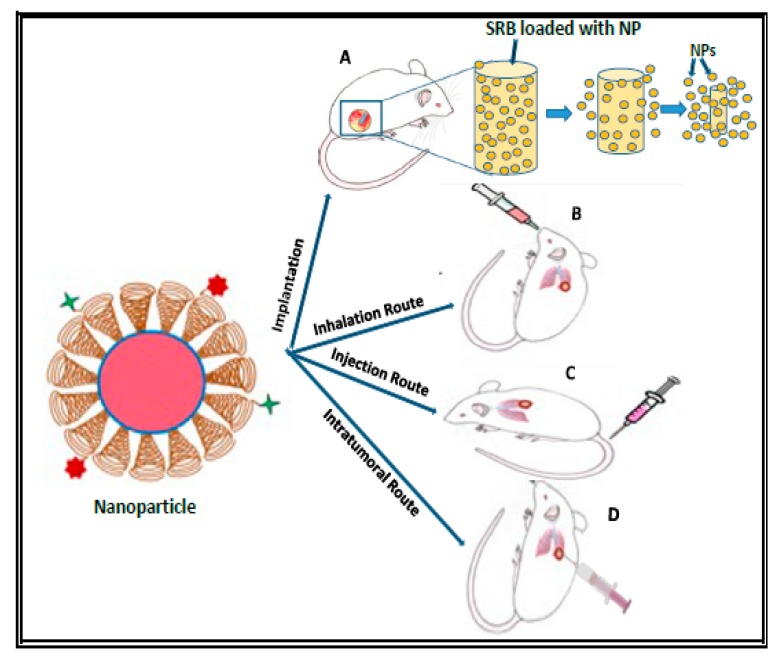
Schematic diagram for current routes of delivery of nanoparticles (NPs). (**A**) Delivery of NPs via implantation (NP-loaded implants such as smart radiotherapy biomaterials (SRBs), e.g., GNP-loaded spacer of length 5 mm and diameter 0.8 mm, similar to current spacers used in RT); (**B**) Delivery of NPs via inhalation (e.g., lung cancer); (**C**) Delivery of NPs via systemic injection (e.g., lung cancer); and (**D**) Delivery of NPs via intratumoral injection (e.g., lung cancer).

Nanoparticle-based radiosensitization is a technique of using nanoparticles (approximately 1–100 nm in size [16]) to increase the susceptibility of tumor cells to ionizing radiation to achieve a higher radiotherapeutic ratio. A radiosensitizer is an agent (e.g., drug, NP, etc.) that causes “radiosensitization”. Thus, radiosensitizers are agents that increase the susceptibility of tumor tissue to injury/damage by quickening DNA damage and creating free radicals when they are subjected to ionizing radiation [17]. In this review, nanoparticles used for radiosensitization are discussed. They can be either therapeutic agents (e.g., drug-based nanoparticles, such as drug-encapsulated polymeric NPs, and platinum-based nanoparticles) or inert therapeutic agents (e.g., gold nanoparticles) that increase the effects of ionizing radiation [1,2,8,10,18,19,20]. The therapeutic NPs (e.g., cisplatin, oxaliplatin or carboplatin NPs) can sensitize and kill cancerous cells without ionizing radiation. They can also act as radiosensitizers and increase the therapeutic ratio when combined with ionizing radiation exposure due to their high atomic numbers (Z) [3,21,22]. Therapeutic NPs can be made by encapsulating or attaching drugs to high-Z nanoparticles such as GNPs [23]. In this case, the drugs become the therapeutic agent, while the high-Z NPs interact with the ionizing radiation for dose enhancement. Meanwhile, interaction of ionizing radiation with high-Z NPs, and the absorption of photoelectrons by high-Z NPs, can result in the release of numerous Auger electrons that are important to the dose escalation of the radiotherapeutic window [4,20,24]. Auger electrons have a lower energy with a shorter range (μm), resulting in deposition of energy in a small proximity within the tumor [25]. However, gold nanoparticles (GNPs), like other high-Z metal-based NPs, are believed to be chemically inert on their own, but are very potent when they interact with ionizing radiation [2,26]. Other studies suggest that the surfaces of GNPs are electronically active and can catalyze chemical reactions, thereby increasing the generation of reactive oxygen species (ROS) [25,27]. The mitochondrial membrane can be oxidized by ROS, disrupting its strength and causing leakage of superoxide anions into the cytosol, resulting in hydrogen peroxide (H_2_O_2_) generation [25]. They are then transported through membranes and damage DNA [25,28]. This process (i.e., biological mechanisms contributing to radiosensitization), combined with ionizing radiation increases the radiosensitization of GNPs [25,28,29]. GNPs have surface properties that can be engineered for specific applications, making GNPs one of the most promising radiosensitizers for RT [17,23]. GNPs can be glycol (PEG)-coated or complexed with other targeting ligands/agents for specific targeting purposes [30,31,32]. Studies have shown that PEG-coated GNPs have improved cellular uptake compared to GNPs without any ligands [30,31]. Metallic nanoparticle-based radiosensitizers are considered as novel enhancers for RT due to their radiosensitizing abilities and synergistic effects on cancerous cells [33]. The high electron density of the metallic nanostructures or NPs plays an important role in attaining a high radiation dose during RT [3,21]. In vitro and in vivo studies, as well as Monte Carlo simulation, have demonstrated increased dose enhancement effects via high-Z NPs [3,18,19,21,34,35,36]. Radiation dose enhancement factor (DEF) increases with increasing NPs concentration in the tumor, as illustrated in Figure 1 [21]. The research interest in nanoparticles and other microparticles has increased rapidly. For instance, in the drug delivery field, dendrimers, micelles, liposomes, nanocrystals, quantum dots, metallic NPs (e.g., gold, gadolinium, titanium, silver, platinum, or hafnium nanoparticles), nanotubes, biodegradable polymeric NPs, lipid NPs, superparamagnetic iron oxide NPs, and nonmetal NPs (e.g., silicon and fullerene) are used as carriers for different drugs (including chemotherapy agents) and other bioactive molecules [8,10,37,38]. This is because NPs can be designed to protect and stabilize the imbedded drug and improve drug delivery at targeted tumor sites [37]. That is, drug efficiency can be increased, while decreasing the side effects [37].

However, despite the significant advancement in the design of NBRs/NPs for RT, delivery of these NPs while avoiding unacceptable side effects of the encapsulated drug as a result of NP interaction with biological systems or cells is of great concern. The potential side effects of radiosensitizing drugs may occur depending on the design/formulation, the technique of NPs delivery, the amount delivered, and the individual patient’s response to the drug [39]. That is, the side effects of the drug and their severity varies from person to person and with drug type. The route of NP delivery can have a substantial impact on its therapeutic or radiosensitizing efficiency. For instance, repeated systemic administration (intravenous injection or infusion) of conventional drug can lead to drug accumulation and thus side effects [39,40]. Therefore, several attempts have been made to employ nanotechnology to enhance the delivery efficiency, increase patient compliance, reduce side effect of drugs, and reduce the frequency of drug dosing required to achieve the desired therapeutic outcomes [15,40,41]. Hence, in-depth understanding of current NP delivery techniques (e.g., systemic or localized delivery) to overcome conventional drug delivery limitations constitutes the major focus of this review. 

The goal of this review was to discuss the delivery techniques of nanoparticle-based radiosensitizers in RT; namely, systemic delivery via injection/infusion and inhalation, as well as localized delivery via NP-implants and intratumoral injection, as shown in Figure 1. The challenges, opportunities, and future prospects of nanoparticle-based radiosensitization (NBR) are highlighted. The enhancement of radiotherapeutic efficacy through NBRs in RT is vital for the killing/control of localized tumors [3,42,43]. However, the efficacy of RT is still restricted by the tolerance of NRBs and radiation dose of healthy tissues surrounding the tumor cells, despite significant improvement in tumor tracking and targeted delivery of both NRBs and radiation to cancerous tissues [3,4,20].

## 2. Delivery Routes of NBRs for RT Applications

Radiosensitizers should be designed to have sensitivity to tumor environment and specific stimuli, such as temperature, pH, magnetic field, light or radiation, and ultrasound intensity. Therefore, delivery techniques/routes of nanoparticle-based radiosensitizers (NBRs) for RT applications play very important roles in tumor sensitization, resulting in radiotherapeutic dose ratio enhancement. Hence, when combining NPs/NBRs with radiotherapy, the therapeutic treatment window should be carefully evaluated to avoid toxicity to healthy tissues [3]. The possible delivery routes discussed here are systemic routes via injection or infusion of NPs, and inhalation route, as well as localized routes via intratumoral delivery, and implantable devices such as implants loaded with NPs or anticancer agents, as shown in Figure 1.

As illustrated in Figure 1, nanoparticles or NBRs can be administered systemically or locally, depending on the selected route and the tumor site. Figure 1 illustrates PEGylation of nanoparticles, which are further conjugated with Alexa Fluor 647 (AF647) carboxylic acid [31]. Poly (ethylene glycol) (PEG) molecules form a layer (indicated by spiral shapes) around the NP (with red blobs indicating an encapsulated drug/agent), which are then bound by AF647 (cross/plus symbol). Local delivery via NP-loaded implants are made from biodegradable polymers (e.g., poly (e-caprolactone), poly (d,l-lactide-co-glycolide) (PLGA), chitosan, etc.), or bioerodable polymers (e.g., polyanhydride) [2,15,44,45,46]. An example of NP-loaded SRBs is a smart spacer specifically designed to release the embedded NPs inside the tumor, and they are meant to replace the inert spacers currently used in RT procedures [2,15,44,47,48]. Several NBRs for enhancing RT have been reported, both in vitro and in vivo, with optimistic outcomes [3]. The choice of delivery route and the interactions of NPs with biological systems (including tumor and normal cells), which depends on the physical and chemical characteristics of NPs [3], contribute to the level of radiosensitization.

### 2.1. Systemic Delivery Routes

NBR administration by systemic routes can have effects on healthy organs despite the concept of “targeted delivery”, which tends to impact the general health of cancer patients, especially for localized solid tumors where there is a need for local radiosensitization [3]. Generally, drugs/nanoparticles can be administered systemically (into human body) via routes such as direct injection/infusion, oral intake, oral inhalation (simply inhalation), and intranasal drug delivery [49,50,51]. In this review, intravenous injection and inhalation delivery routes are discussed. 

**Figure 2:**
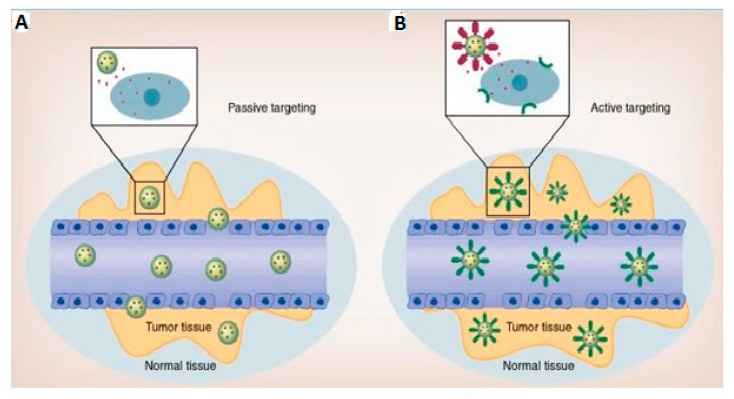
Illustration of passive and active targeting of nanoparticles (NPs) for enhancing the therapeutic efficacy of anticancer drugs (obtained permission from Weihong Tan, via email correspondence, reference [53]); (**A**) Passive targeting of NPs taking advantage of the enhanced permeability and retention (EPR) effect, and (**B**) Active targeting of NPs attached with ligands to enhance accumulation and cellular uptake of NPs via receptor-facilitated endocytosis [53].

The best strategy to improve the systemic delivery of NBRs is by employing targeted NBRs (NPs that precisely target anticancer drugs to cancerous cells) [52,53]. After administration, the NBRs reach the desired tumor site with marginal loss in blood circulation, and the NBRs can act on the targeted tumor cells with reduced side effects on normal tissues [52,53]. The two main strategies, as shown in Figure 2, that have been embraced by researchers for NP/drug targeting are passive targeting and active targeting [52,53].

Passive targeting, as seen in Figure 2A, exploits the distinctive pathophysiological conditions of the target tumor vessels, such as leaky vasculature [53].

Tumor vessels are usually extremely disordered and dilated with pores, causing gaps between endothelial cells, and thereby compromising lymphatic drainage [53]. The “leaky” vascularization is referred to as the enhanced permeability and retention (EPR) effect, allowing macromolecules of diameter up to 400 nm into the neighboring tumor tissue [53,54,55,56]. Some researchers believe that NPs can accumulate within tumors due to the EPR effect while avoiding most normal cells [41,56]. Passively targeted NPs can be specifically designed as a result of the EPR effect [20,37,57,58,59]. Although the passive targeting technique is clinically accepted, it has numerous limitations, such as inefficient NP/drug diffusion, the random nature of NP circulation in the blood prior to reaching the tumor environment, and the fact that some tumors do not display an EPR effect, and the penetrability of vessels are unlikely to be uniform in a single tumor [53,55,60,61]. 

On the other hand, active targeting of NPs is a technique that some scientists believe can overcome the limitations of passive targeting [53]. In active targeting, illustrated in Figure 2B, the surfaces of the NPs are attached with ligands (peptides, antibodies, aptamers, or other small molecules) by a range of chemical conjugations that can bind specifically to receptors on the surface of cancerous cells [53,55,62]. Nevertheless, actively targeted NPs are required to reach the targeted tumor site in order to bind to the receptors and antigens on the cancerous cells [55]. The selected ligands meant to bind to overexpressed/clustered receptors on the tumor cell surfaces are intended to increase the uptake and retention by the targeted tumor cells [55,63]. To achieve optimum delivery of actively targeted NPs, receptor and antigen must be abundantly present on the tumor surface, and the ligand–antibody pair must have a high affinity in order to bind to the overexpressed cell surface molecules [63]. While the active targeting approach has great potential, the NPs/drugs presently accepted for clinical use are somewhat simple and are mostly incapable of activating drug release mechanisms [53].

Whereas passively targeted NPs utilize the EPR effect for targeting cancerous cells, actively targeted NPs’ surfaces are attached with ligands in order for the NPs to bind to the receptors that are overexpressed by endothelial cells or cancerous cells [53]. Some passively targeted NPs have been accepted for clinical use; however, no actively targeted NPs have successfully passed clinical trials for clinical use [55]. 

#### 2.1.1. Delivery of NBR via Intravenous Routes

In the traditional intravenous routes (injection or infusion) of delivery of NPs, the NPs have to navigate through physiological barriers before some portion of the NPs reaches the tumor site [1]. Studies have shown that of the NPs delivered (including targeted drug and nanoparticles) via systemic routes, only a portion of the administered dosage reaches the target tumor site, even with the enhanced permeability and retention (EPR) effect [3,57,64,65], whereas the remainder of the dose remains in systemic circulation, leading to systemic toxicity [57]. For instance, an in vivo study in mice indicated that intravenous (IV) injection of fluorescent PLGA-NPs of sizes 200 nm and 500 nm were accumulated highest in the liver, spleen, and lungs [37]. The therapeutic efficacy and outcome of NBRs also depend on the blood circulation time [66]. NBRs with long circulation times have the benefit of increased probability of accumulation in the tumor-microenvironment. After extravasation from the blood vessels, permeation into deep tumor cells becomes challenging due to poor lymphatic drainage and high interstitial fluid pressures in the tumor volume [66,67]. However, NPs/NBRs with short release of the encapsulated drug are not good candidates for systemic delivery since the drug can release prior to reaching the desired tumor site. As a result, other delivery routes, such as inhalation, tumor injection, and local implant delivery, have been exploited.

#### 2.1.2. Delivery of NBRs via Inhalation

**Figure 3:**
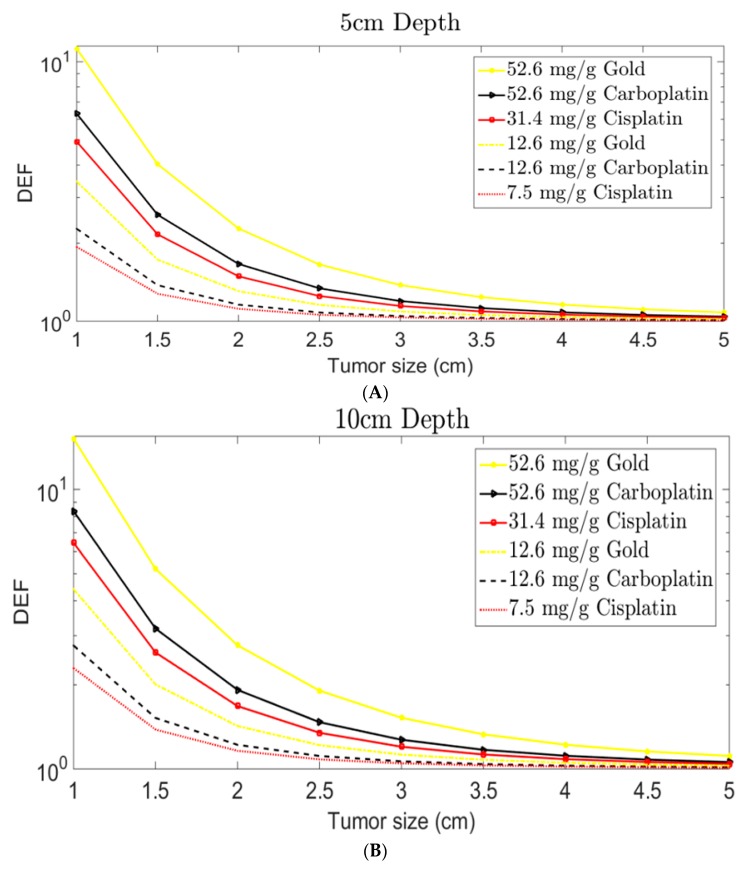
Illustration of NPs delivered via inhalation for tumor radiation dose enhancement as a function of tumor size at different concentrations for carboplatin, cisplatin, and gold nanoparticles, respectively (reference [68]); (**A**) DEF at 5 cm depth; (**B**) DEF at 10 cm depth.

Delivery of NBRs via inhalation is a technique in which tumors, such as lungs cancers, can be radiosensitized with NBRs. Lung cancers are among the leading cause of cancer-associated mortality and generally have short survival rates of about 16% within five years [68,69]. However, radiation boosting can substantially increase the survival rate for patients with lung cancers, but is restricted by healthy tissue toxicity due to the respiratory motion [68,70,71,72]. The proposed treatment strategy to overcoming the limitations is to increase the dose to the tumor volume, while carefully sparing the surrounding normal lung tissue by increasing local radiosensitization via inhalation nanoparticles, as seen in Figure 1B, and decreasing the dose to balance complications and cancer cure [1,68]. Combined chemoradiotherapy is the best strategy for boosting the radiation dose to the tumor. However, studies have shown that only about 5% of NPs administered via intravenous (IV) routes reach the lungs [1,68,73]. Conversely, delivery of NPs via inhalation leads to the accumulation of NPs in the lungs, limiting the escape of the NPs into the systemic circulation, thereby limiting systemic toxicity [73]. Therefore, delivery of NPs via inhalation can enhance radiation dosage, with minimal toxicities to normal tissue. Delivery via inhalation of gold nanoparticles (GNP), cisplatin nanoparticles (CNP), and carboplatin nanoparticles (CBNP) has been investigated [68]. It was found that delivery of NPs via inhalation can result in 3.5 to 14.6 times higher concentrations than ordinary IV delivery [1,68]. It was also reported that the DEF increases with increasing field size or decreasing tumor volume, as shown in Figure 3 for inhalation of NPs during combined chemoradiotherapy with 6 MV external beam RT [68]. Studies have shown that the inhalation route offers a more promising method for targeting lung tumor cells with nanoparticles/radiosensitizers, increasing the radiation damage to lung tumors cells, while decreasing the damage to disease-free lung tissue [1]. However, inhaled NPs can be absorbed rapidly in the lungs and can not only act locally, but also systemically. 

### 2.2. Local Delivery Routes

Systemic delivery of NBRs, no matter the effectiveness, requires a high dosage with repeated dosing over a long period to achieve the desired radiosensitizing concentrations at the tumor site [40], thereby increasing the possibility of tumors developing resistance and increasing potential side effects [40]. Factors associated with tumors, such as high interstitial fluid pressure and rigid extracellular matrix, are among the major obstacles to the diffusion of drugs and antibodies (even small molecular weight drugs) [74]. Targeted NPs/drugs delivered by systemic routes have the prospect of reaching the target site at only a small fraction (less than 1%, from recent study [75]) of the administered dosage, even with the EPR effect [55,57]. Unlike the systemic delivery route which is plagued with many limitations, localized NBR delivery can be achieved following surgical and/or nonsurgical procedures [76]. Local delivery does not necessarily require surgical operation; however, it can be administered during surgery and/or routine radiotherapy procedures and does not require surgery for removal after NPs/drug delivery [15,45]. NP-loaded or drug-embedded implants such as eluting spacers are made from bioerodable or biodegradable polymers, which do not require surgery for removal [15,44,46,77]. These polymers degrade into biocompatible compounds that are easily absorbed in the body with virtually no side effects [2,15,44,45,77]. Local NRB delivery can be advantageous, particularly in tumor sites where conventional systemic delivery routes fail [40]. Local delivery routes also offer high NBR concentrations at the targeted tumor volume, eliminate repeated dosing, and have decreased required dosage, decreased toxicity, and higher patient compliance [15,40,41,44].

#### 2.2.1. Intratumoral Delivery

**Figure 4:**
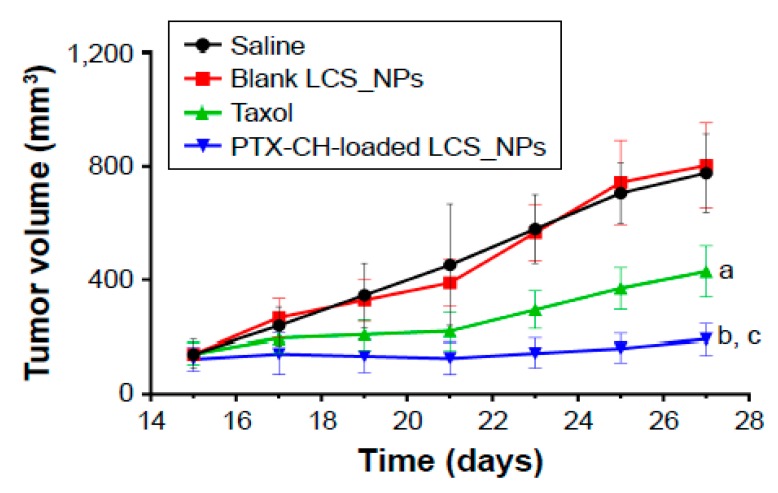
Illustration of intratumoral injection in mice tumor treated with saline, blank lecithin–chitosan nanoparticles (LCS_NPs), PTX, PTX–CH-loaded LCS_NPs (mean ± SD, *n* = 6); where a, *p* < 0.001; b, *p* < 0.001 vs. saline; c, *p* < 0.001 vs. PTX [83].

Direct intratumoral injection (ITJ) of anticancer drugs and NPs radiosensitizers, as seen in Figure 1D, has been broadly assessed for decades and is considered to be a safe and effective treatment route [78,79,80,81,82]. Studies have shown that ITJ delivery may limit systemic toxicity since it can be combined clinically with surgery [81]. ITJ delivery of radiosensitizers is considered minimally invasive due to clinical advances, such as fine needle biopsy, and drug delivery and delivery systems that offer more effective, safer, and nonsystemic delivery methods [81]. Studies have shown that ITJ can significantly improve drug/NPs concentration in the tumor in comparison to systemic IV administration [78]. For instance, Chu et al. prepared paclitaxel–cholesterol-complex-loaded lecithin–chitosan nanoparticles (PTX–CH-loaded LCS_NPs), and assessed the antitumor efficacy of the NPs both in vitro and in vivo for palliative intratumoral injection therapy [83]. These PTX–CH-loaded LCS_NPs were found to inhibit tumor growth and metastasis efficaciously; on average, the survival time of tumor-bearing mice was prolonged, as shown in Figure 4 [83]. Palliative intratumoral injection of chemotherapy in diverse formulations, such as nanoparticles, hydrogel/gel [84,85], microspheres [86], and nanofibers [87], have been used in the treatment of midcervical carcinoma [83,88], laryngeal squamous cell carcinoma [89], recurrence of head and neck squamous cell carcinoma [90,91], and lung cancers with the aid of bronchoscopic endobronchial ultrasound [92,93], among other studies. However, the ITJ delivery route has not been considered as a major route of drug administration in regular clinical practice because further investigation is required [78,80]. 

In general, ITJ is considered to be invasive depending on the tumor site, and there is relatively rapid clearance of the drugs/NPs from the tumor volume into systemic circulation, which could lead to drug toxicity in surrounding tissues [78]. Furthermore, most tumors accessible by ITJ are usually treated with regular and more effective locoregional treatment techniques, such as surgery and radiotherapy [78], or chemotherapy and radiotherapy [6,94,95,96,97]. 

#### 2.2.2. Delivery of NBRs via Implants 

**Figure 5:**
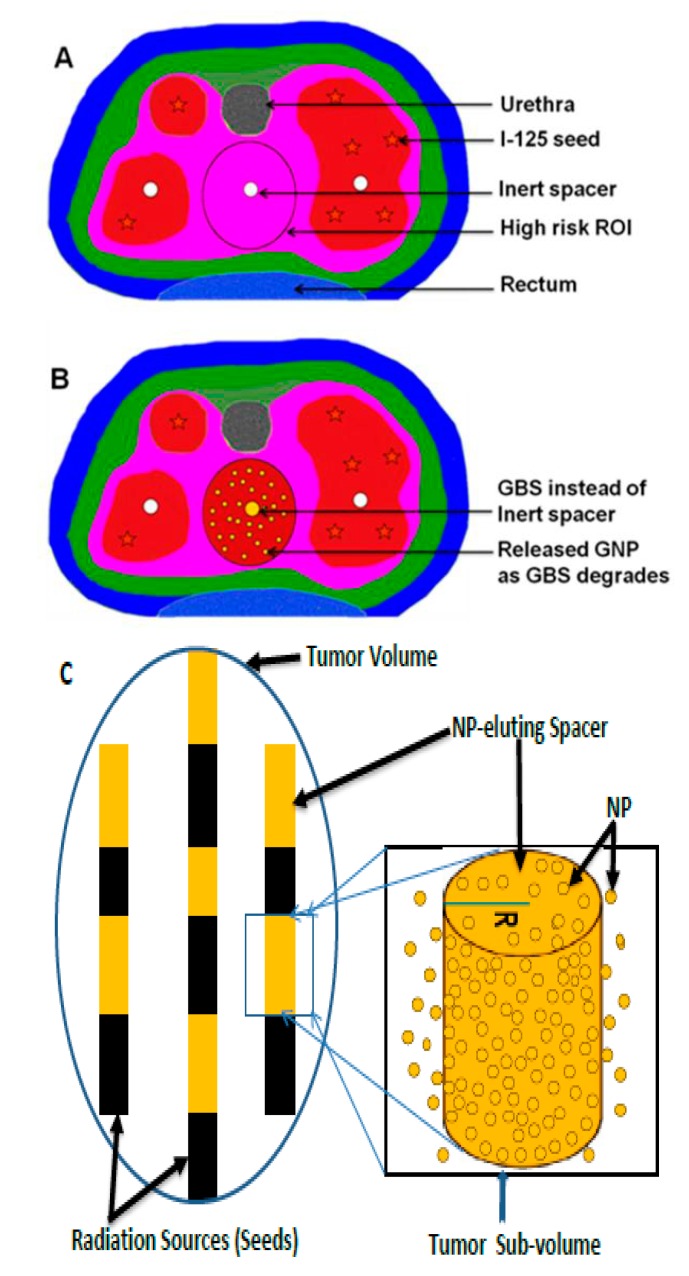
Illustration of the application of SRBs in low-dose brachytherapy of prostate cancer; (**A**) Current routinely used inert spacers in brachytherapy using iodine-125 (I-125), ROI—region of interest [45]; (**B**) New-generation brachytherapy spacer coated with gold nanoparticles (e.g., GNP-loaded brachytherapy spacers (GBS) [45]) instead of inert spacers; (**C**) Schematic illustration of using NP-eluting spacers of radius R to replace inert spacers.

Implants (e.g., millirods, film, wafers, gels, depots, drug-coated stents, etc.) are made from biodegradable or bioerodible polymers loaded with anticancer drugs. They have been used as another medium for direct drug delivery inside the tumor volume [46,98,99,100,101,102,103]. Similarly, smart radiotherapy biomaterials (e.g., NP-loaded spacers, fiducial markers, or hollow spacers loaded with NBRs) are made from biodegradable and bioerodible polymers loaded with NBRs for RT dose enhancement instead of current brachytherapy spacers, as shown Figure 1 and Figure 5 [2,15,21,45,47,48,104,105,106]. This requires no additional procedure since brachytherapy spacers and fiducial markers are routinely used in RT [15,45,48]. NBRs/nanoparticles are loaded on/in SRBs, e.g., via GNP-loaded spacers, as shown Figure 5B,C, for localized delivery inside the tumor volume. The application of smart radiotherapy biomaterials (SRBs) has been considered as a novel approach to increase the radiotherapeutic ratio without requiring additional protocols and is envisioned to replace conventional inert radiotherapy biomaterials (IRBs) [2,15,45]. In conventional RT, IRBs (such as spacers, fiducial markers, applicators, etc.) are used routinely in RT for disease treatment, spatial accuracy, tumor targeting with RT, tracking tumor motions, and guiding robotic radiosurgery [2,4,15,45,104,107,108,109]. However, these IRBs do not offer radiotherapeutic benefits except the primary functions mentioned above [4,15,48,104,106]. Figure 5A is an example of a prostate cancer brachytherapy procedure with application of an inert spacer routinely used in RT [45]. Current studies have shown that SRBs can be used instead of IRBs to perform the primary functions of IRBs while eluting the anticancer agents (e.g., drug, NPs, etc.) embedded in the SRBs to enhance therapeutic efficiency [4,15,44,48,104,105,106], as shown in Figure 5B,C. Therefore, localized in situ delivery of NPs via implants, such as SRBs, is another strategy for tumor radiosensitization to boost radiation dose to the tumor and minimize toxicity to healthy tissues. This is accomplished by replacing inert radiotherapy biomaterials (e.g., spacers, fiducials, applicators, etc.) routinely used in radiotherapy with smart ones (SRBs) loaded with NPs for sustained local release inside the tumor, as shown in Figure 1A and Figure 5 [2,4,15,44,105], while eliminating/reducing radiation toxicity and the systemic effect of intravenous administration of the NPs, as shown in Figure 1B,C. When the SRBs or NP-eluting spacers are inserted during regular RT (external beam RT or low dose brachytherapy), the embedded NPs begin to release upon contacting biological fluid within the tumor as the polymer erodes or degrades. The released NPs or anticancer drugs begin to make the tumor cells more radiosensitive, while the interaction of the NPs with ionizing radiation increases the dose to the tumor [15,21,45]. 

**Figure 6:**
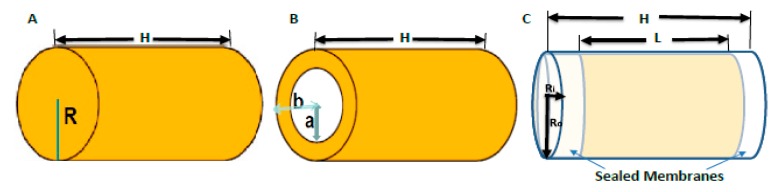
Schematic diagram of current smart radiotherapy biomaterials; (**A**) Coated solid spacer of length H and radius R, fabricated spacers from polymer–nanoparticles mixture/gel; (**B**) Uncoated spacer of length H, inner radius *a*, (i.e., conventional inert spacer coated with NPs), and outer radius *b*; (**C**) Hollow spacers of length H, outer radius *R_o_*, length of inner hollow area L, and inner radius R_i_, loaded with NPs/anticancer agents.

**Figure 7:**
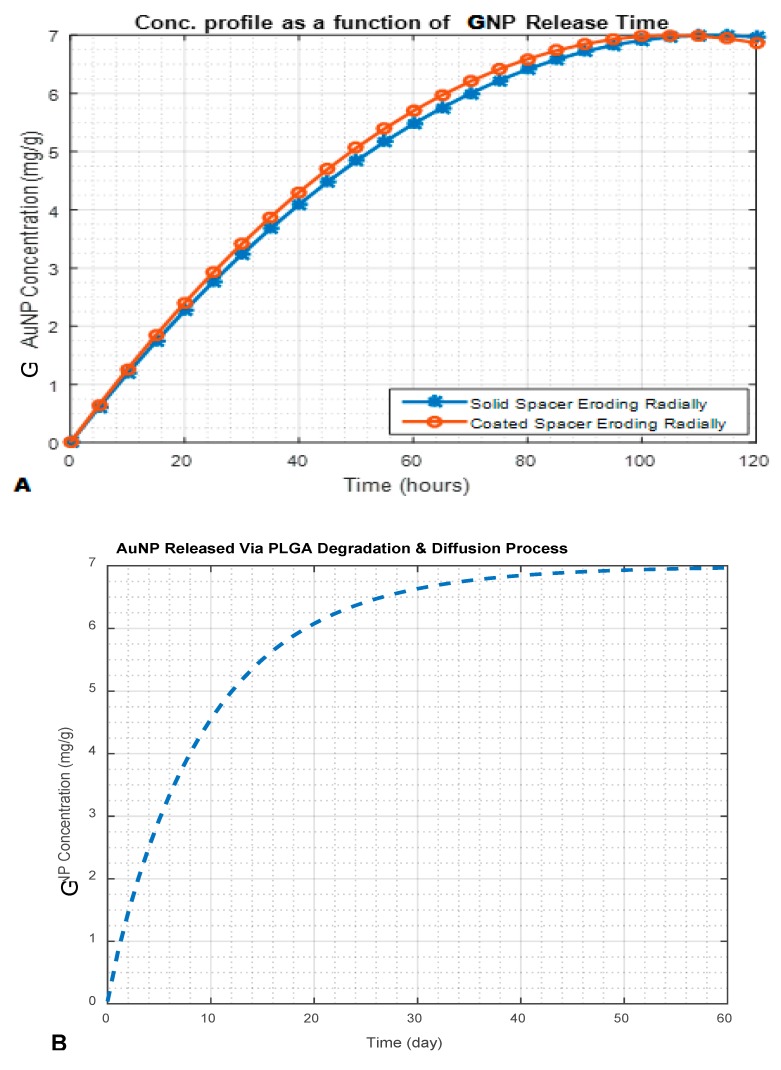

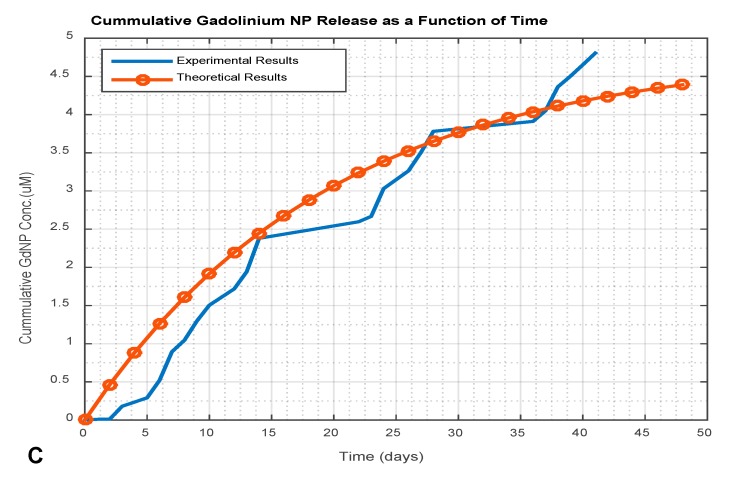
NP release profile for different eluting spacers with 10 nm AuNP; (**A**) 7 mg/g Gold NPs releasing from erodible spacers (comparing coated erodible spacer to solid erodible spacer); (**B**) 7 mg/g Gold NPs releasing from PLGA (degradable polymer) spacers loaded with AuNP; (**C**) 4.81 µM gadolinium nanoparticles (GdNP) releasing from hollow spacers (comparing in vitro experimental results and a theoretical model, explained in detail in [2,47]).

These new-generation SRBs (brachytherapy spacers, fiducials, hollow spacers, etc.) can be prepared based on the type of SRB and the intended application. Coated spacers are made by coating the conventional spacer with a NPs/drug–polymer mix/gel and kept dried, as shown Figure 6B and Figure 7A for a coated erodible spacer [44]. Uncoated solid spacers are fabricated from the NPs/drug–polymer gel/mixture as shown in Figure 6A and Figure 7A for solid erodible spacers [2,44]. Hollow spacers, as shown Figure 6C and Figure 7C, have a hollow area inside the spacer that is filled with NPs/drug [2,47,105]. Hollow spacers are considered multifunctional smart radiotherapy biomaterials, since they can be loaded with different cargo (NPs, drugs, or anticancer agents), for multipurpose applications in RT, MRI, etc., and can also be tailored to release the cargo at release rates shorter than coated and solid spacers [2,47,105]. Figure 7 demonstrates NP release from NP-eluting spacers for both in vitro experimental results and theoretical mathematical models (details are discussed by Boateng, F., and Ngwa, W. in their previous published papers [2,15,44]). Figure 7A shows NP release of less than 120 h for erodible spacers, whereas Figure 7B shows NP release from a biodegradable spacer that took more than a month (about 52 days) [44]. Thus, for spacers of the same dimensions, bioerodible spacers have a faster release rate than that of biodegradable spacers. Therefore, erodible spacers are good candidates for short (e.g., Pd-103) and long lived (e.g., I-125) radioisotopes, unlike degradable spacers, which are only good for long-lived radioisotopes [44]. However, Figure 7C shows the release (~41 days) of gadolinium nanoparticles (GdNP) from hollow spacers (made from PLGA). Therefore the NP release from spacers depends on the NP type, the type of polymeric material used, the ratio of the NPs/drug to the polymer, the number of spacers used, and the release medium (tumor microenvironment) [15]. 

**Figure 8:**
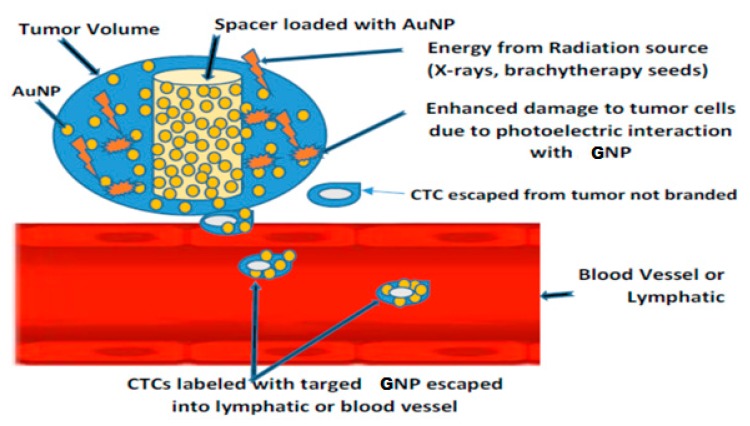
Labeling and tracking circulating tumor cells (CTCs) using SRB loaded with targeted gold nanoparticles (GNP). The labeled CTCs can be detected by MRI or PAI [2].

Figure 8 illustrates the potential for SRBs loaded with targeted gold nanoparticles (GNP) to label tumor cells during radiotherapy to track labeled circulating tumor cells (CTCs), which are a biomarker of cancer metastasis [2,110,111]. Cancer metastasis is caused by CTCs which are shed from the principal tumor into the lymph nodes or blood vessels [112,113]. CTCs labeled with NPs can be detected or captured via noninvasive procedures, such as magnetic resonance imaging (MRI), photoacoustic imaging (PAI), or flowmetry [114,115,116,117,118,119,120,121,122,123], without drawing blood samples, in contrast to the conventional procedures with low sensitivity due to small blood volumes (5–10 mL) that are usually used [112]. Studies have shown that about one to ten CTCs can be found in 1 mL of blood [112,124]. Therefore, SRBs loaded with GNPs can have a dual purpose of dose enhancement and tumor cell labeling without compromising RT procedures [2,110,111]. 

## 3. Distribution of NBRs in Tumor Volume

Tumor penetration of NBRs and the drug release from drug-encapsulated NPs in the tumor volume correlate to the extent of radiosensitization [125]. Drug distribution in the tumor has been recognized as one of the greatest challenges in drug delivery [126]. Understanding the physiology of various cancers and NPs/drug transport in the tumor is essential for NBRs. NPs or the embedded drug cannot do any good unless the NPs reach the target tumor and the NPs with embedded drug release their content [126]. Barriers (varying by cancer type), such as high interstitial fluid pressure, dense extracellular matrix, increased diffusional distances, high cell density, as well as the general heterogeneity of the tumor cells, should be overcome to achieve higher NPs/drug concentration in the tumor [126,127]. Obtaining accurate measurements of NPs/drug within specific tumors is another hurdle [126]. The physicochemical properties of NPs, including shape, size, surface chemistry (e.g., PEGylation or conjugation), surface charge, affinity, and composition (material hydrophobicity or hydrophilicity) affect the intratumoral penetration, pharmacokinetics, biodistribution, and bioavailability of the NBRs in the tumor [74,125,128,129,130]. However, tumor biology (such as perfusion, blood flow, interstitial fluid pressure, permeability, and stroma content) and the characteristics of the patient (e.g., gender, age, tumor location in the body, tumor type, body composition, and past treatment history and modality) influence the delivery of drug via nanoparticles [125].

With the aid of improved imaging techniques, studies have shown the biodistribution of NPs in animal models [74,129,131]. The postextravasation outcome of NPs varies with the physiochemical properties of the NPs [74]. Studies have also shown that NPs with cell-specific ligands have high retention in tumors compared to NPs without ligands, since ligands have an influence on the retention and distribution of NPs in the tumor volume [74,132]. For instance, Kong T. et al. reported that functional thioglucose (Glu) gold nanoparticles (Glu-GNPs) showed significantly higher cancer cell uptake than bare GNPs [133]. However, the tumor-specific ligands may obstruct the penetration of the NPs into the tumor volume [74]. Targeted nanoparticles (regardless of the existence of tumor-cell-specific ligands on the NP surface) generally accrue in solid tumors through the compromised lymphatic drainage and the permeable vasculature, depending on the circulating period [59,74]. 

NPs of smaller size have a larger surface area-to-volume ratio; therefore, most of the embedded drug is at or near the surface, leading to faster drug release from small NPs [134]. The setback is that smaller drug-loaded NPs are administered via the systemic route with longer circulating time, which could lead to release of their content before reaching the tumor. Larger NPs have larger cores, and more drugs can be embedded per particle; however, they have slower release rates compared to smaller NPs [134]. Thus, drug release from the NP can be controlled as a function of NP size [134]. Smaller NPs have greater ability to penetrate tumors than larger NPs. However, studies have shown that different types of NPs exhibit different penetration abilities in tumor tissue [130,135]. Fast intracellular uptake of NPs significantly enhances tumor targeting, leading to high local drug/NP concentrations [129]. However, it is reported that both the NPs and the tumor microenvironment can be optimized or modulated for optimum delivery of NPs [125,136,137].

## 4. Challenges and Opportunities in NBR for RT

The application of NPs for radiosensitization in RT is growing; however, there are challenges and opportunities that require extra research and development for clinical applications [4]. The challenge of delivering sufficient and potent concentrations of NPs to the tumor volume with negligible side effects is essential for improving therapeutic efficacy [1,20]. Current administration via systemic routes (off-target delivery or “sea” delivery) is considered to have adverse effects on organs other than tumor cells, thereby decreasing the general health of the patients, especially where localized radiosensitization is needed [1,3]. Henceforth, the therapeutic window of combining nanoparticle-based radiosensitizers with radiotherapy should be cautiously evaluated for toxicity of radiosensitizers on healthy tissues [3]. Another challenge associated with systemic delivery is that NPs with prolonged circulating time might not reach the tumor site during a radiotherapy procedure. Delaying treatment may or may not contribute significant dose enhancement in comparison to localized delivery. Current understanding of active targeting of NPs with ligands, or passive targeting of NPs via the EPR effect and their effect on NPs penetration, uptake, and accumulation in tumor cells is typically based on mouse models (fast-growing xenografted mice with dense vasculature), which do not replicate the majority of human solid tumors [55]. Further investigations are necessary to improve the current strategies of targeted NPs for systemic delivery.

Among the routes of delivery, NPs delivery via implants/SRBs is the safest delivery route, with no/less systemic toxicity, compared to the inhalation route. SBRs are routinely used in RT, and therefore require no additional special procedure for implantation or surgical removal after implantation [4,15,45]. However, application of SRBs could be limited to specialized cancers, such as prostate, breast, lungs, or areas where conventional spacers and fiducial markers are routinely used. Likewise, the inhalation route might be effective only for lung cancers.

The route of delivery of the NPs and the complexities of the material used for the preparation of the NPs or delivery vehicle (NPS, implants, etc.) present challenges depending on the tumor site. As effective as smart implants, such as SRBs, polymeric materials (biodegradable or bioerodible/bioerodable polymers) employed for designing these SRBs to deliver the embedded radiosensitizers within therapeutic window for external beam RT or brachytherapy application should be considered [2,15,44,47]. Therefore, the time taken for the NPs to release from SRBs should be accounted for during treatment planning [2,15,44]. 

The challenge of polymeric drug-loaded NPs capable of retaining the drug content until reaching the tumor site (if a systemic delivery route is used) should also be noted. Any leakage or burst release before reaching the desired site will result systemic toxicity, reducing the NPs/drug concentration in the tumor volume [138]. The effect of an initial burst release of radiosensitizing drugs encapsulated in the NPs is a major challenge of systemic delivery of radiosensitizing NPs for RT applications. The fast release of drug (in an early burst stage) has negative effects, which can be pharmacologically precarious [139]. Therapeutic or radiosensitizing drug release during the early stages depends on the diffusion of the escaped drug from NPs (especially polymer drug-encapsulated NPs), and polymeric degradation/erosion before reaching the desired tumor sites. Therefore, initial burst release of the encapsulated drugs/agents from such NPs should be prevented or reduced [140,141,142]. The initial burst release of drug from NPs results from poor drug encapsulation and fabrication/formulation techniques (e.g., emulsion–solvent evaporation or extraction technique) [140,143]. Hence, there should be high drug encapsulation efficiency and the formulation parameters for making NPs should be considered [140,142], creating diffusional resistance and preventing the quick dissolution of the drug into blood circulation [141]. The physical and chemical properties of the encapsulating polymers, the solvents used, the drug types (hydrophobic or hydrophilic), and drug–polymer interactions should be taken into account during fabrication of NPs for radiosensitization in RT applications [140,143].

The combination of diverse hydrophobic moieties and hydrophilic polymeric backbones that have potent in vivo distributions in the tumor is recommended [129]. For example, Cho Y, et al. reported the use of fluorescein isothiocyanate-conjugated glycol chitosan nanoparticles that displayed highly selective tumor localization [129], essential for imaging modalities. Therefore, designing radiosensitizers with highly selective tumor localization can play an important role in achieving effective radiation dose enhancement. Radiosensitizers are promising radiotherapeutic agents for enhancing damage to tumor cells by speeding-up DNA damage of tumor cells and producing free radicals, yet strategies (including research and preclinical studies) to design low toxicity and more highly effective radiosensitizers are still needed [1,17]. Nevertheless, the predictability of the pharmacokinetics of the radiosensitizers and timing of delivery of the administered NPs in the tumor with radiotherapy treatment should be paramount considerations.

## 5. NBR in Perspective

There is a great prospect for the application of nanoparticle-based radiosensitization in RT, especially the application of SRBs for in situ dose-painting in RT [2,4,44,45,105]. Brachytherapy Application with In-situ Dose-painting via Gold Nanoparticle Eluters (BANDAGE) is a new approach proposed to increase therapeutic efficacy during brachytherapy [2,4,15,44]. The highest clinical impact of BANDAGE is anticipated to significantly increasing the survival and quality of life for prostate cancer patients who require salvage therapy but have reached their normal tissue radiotherapy dose limits [2,4]. If successful, it is envisioned that BANDAGE would also be used during initial prostate-seed brachytherapy treatment to boost local radiotherapy effect without increased toxicity, helping to prevent prostate cancer recurrence [2,4].

However, the addition of new sophisticated molecular targeting agents to the radiosensitizers/NPs would lead to higher radiotherapeutic ratios [95,144]. Nanoparticle-based radiosensitizers for radiotherapeutics are in a position to play a very significant role in the progress of cancer treatments, including concomitant chemoradiotherapy [95]. The ultimate goal of nanoparticle-based radiosensitizers is to achieve greater curing, lower systemic toxicity, and reduced tumor reoccurrence, thereby prolonging and improving cancer patients’ lives [95]. The ideal platform would be when radiosensitizers can reach tumor sites in sufficient concentrations and selectively act in the tumor volume without toxic effects on normal tissues. The ideal radiosensitizer does not exist currently [6]; however, with an improved delivery route (such as via SRBs), significant dose enhancement can be achieved with minimal radiation dose toxicity and minimal systemic toxicity of drugs and anticancer agents.

## 6. Conclusions

Nanoparticle-based radiosensitization, as a potential tool for radiation dose enhancement, presents an ideal means to increase the ionizing radiation dose within the tumor volume with reduced energy (radiation dose) delivery, while sparing healthy tissues surrounding the tumor. The nanoparticles should be designed and evaluated extensively in nonclinical or preclinical studies to ensure the effective translation from research laboratories to clinics with evidential proof of efficacious enhancement validated in preclinical and clinical trials. The ideal radiosensitization would have marginal systemic toxicity, higher enhancement, and negligible radiation dose toxicity, thereby prolonging cancer patients’ lives after treatment.

## Abbreviations

DEF	Dose enhancement factor
GBS	GNP-loaded brachytherapy spacer
GNP	Gold nanoparticle
NBRs	Nanoparticle-based Radiosensitizers
CTCs	Circulating tumor cells
IRBs	Inert radiotherapy biomaterials
ITJ:	Intratumoral injection
LCS_NPs	Lecithin–chitosan nanoparticles ROI: Region of interest
MRI	Magnetic resonance imaging
PAI	Photoacoustic imaging
PEG	Poly(ethylene glycol)
ROI	Region of interest
SRBs	Smart radiotherapy biomaterials
